# Salvage Nodal Radiotherapy as Metastasis-Directed Therapy for Oligorecurrent Prostate Cancer Detected by Positron Emission Tomography Shows Favorable Outcome in Long-Term Follow-Up

**DOI:** 10.3390/cancers14153766

**Published:** 2022-08-02

**Authors:** Jörg Tamihardja, Leonie Zehner, Philipp Hartrampf, Dominik Lisowski, Susanne Kneitz, Sinan Cirsi, Gary Razinskas, Michael Flentje, Bülent Polat

**Affiliations:** 1 Department of Radiation Oncology, University of Wuerzburg, 97080 Wuerzburg, Germany; leoniezehner@gmx.de (L.Z.); lisowski_d@ukw.de (D.L.); cirsi_s@ukw.de (S.C.); razinskas_g@ukw.de (G.R.); flentje_m@ukw.de (M.F.); polat_b@ukw.de (B.P.); 2 Department of Nuclear Medicine, University of Wuerzburg, 97080 Wuerzburg, Germany; hartrampf_p@ukw.de; 3 Biochemistry and Cell Biology, Biocenter, University of Wuerzburg, 97074 Wuerzburg, Germany; susanne.kneitz@uni-wuerzburg.de

**Keywords:** metastasis-directed therapy, long-term outcome, oligorecurrence, prostate cancer, salvage radiotherapy, PSMA

## Abstract

**Simple Summary:**

Patients, who suffer from oligorecurrent prostate cancer with limited nodal involvement, may be offered positron emission tomography (PET)-directed salvage nodal radiotherapy to delay disease progression. This current analysis aimed to access salvage radiotherapy for nodal oligorecurrent prostate cancer with simultaneous integrated boost to PET-involved lymph nodes as metastasis-directed therapy. A long-term oncological outcome was favorable after salvage nodal radiotherapy and severe toxicity rates were low. Androgen deprivation therapy plays a major role in recurrent prostate cancer management and demonstrates a positive influence on the rate of biochemical progression in patients receiving salvage nodal radiotherapy. The present long-term analysis may help clinicians identify patients who would benefit from salvage nodal radiotherapy and androgen deprivation therapy, as a multimodal treatment strategy for oligorecurrent prostate cancer.

**Abstract:**

Background: The study aimed to access the long-term outcome of salvage nodal radiotherapy (SNRT) in oligorecurrent prostate cancer. Methods: A total of 95 consecutive patients received SNRT for pelvic and/or extrapelvic nodal recurrence after prostate-specific membrane antigen (PSMA) or choline PET from 2010 to 2021. SNRT was applied as external beam radiotherapy with simultaneous integrated boost up to a median total dose of 62.9 Gy (EQD2_1.5Gy_) to the recurrent lymph node metastases. The outcome was analyzed by cumulative incidence functions with death as the competing risk. Fine–Gray regression analyses were performed to estimate the relative hazards of the outcome parameters. Genitourinary (GU)/gastrointestinal (GI) toxicity evaluation utilized Common Toxicity Criteria for Adverse Events (v5.0). The results are as follows: the median follow-up was 47.1 months. The five-year biochemical progression rate (95% CI) was 50.1% (35.7–62.9%). Concomitant androgen deprivation therapy (ADT) was adminstered in 60.0% of the patients. The five-year biochemical progression rate was 75.0% (42.0–90.9%) without ADT versus 35.3% (19.6–51.4%) with ADT (*p* = 0.003). The cumulative five-year late grade 3 GU toxicity rate was 2.1%. No late grade 3 GI toxicity occured. Conclusions: Metastasis-directed therapy through SNRT for PET-staged oligorecurrent prostate cancer demonstrated a favorable long-term oncologic outcome. Omittance of ADT led to an increased biochemical progression.

## 1. Introduction

Metastasis-directed therapy (MDT) for oligometastatic disease has gained increasing support for different entities recently [1,2,3]. For prostate cancer, stereotactic ablative radiotherapy (SABR) has been proven to be efficient in controlling oligometastatic disease, and it has resulted in an increased progression-free survival and androgen deprivation therapy (ADT)-free survival in the ORIOLE and STOMP trials, respectively [4,5].

To identify patients with oligometastasis who would benefit from localized therapy, advanced molecular imaging methods are crucial [6]. Although the first MDT trials in prostate cancer utilized choline positron emission tomography (PET), prostate-specific membrane antigen (PSMA) PET represents the current gold standard for detecting metastatic disease [4,7]. PSMA PET allows the detection of small lymph node metastases even in men with early prostate-specific antigen (PSA) value progression (≤0.5 ng/mL), resulting in improved patient selection and the early diagnosis of nodal oligorecurrent prostate cancer [8,9].

Although the first trials showed promising data for MDT in oligometastatic prostate cancer, no consensus regarding the optimal treatment regime for nodal oligorecurrent prostate cancer yet exists [4,5,10,11,12]. Albeit offering high local control, SABR suffers from nodal out-of-field recurrences, which was highlighted by a multicenter analysis by De Bleser et al. [13]. In their analysis of 506 patients, three-year metastasis-free survival was worse with SABR than with elective salvage nodal radiotherapy (SNRT) with 68% versus 77%, respectively [13]. The Prostate Cancer Expert Panel of the German Society of Radiation Oncology (DEGRO), therefore, preferentially recommended elective salvage nodal radiotherapy with simultaneous integrated boost (SIB) to involved lymph nodes for pelvic nodal oligorecurrent prostate cancer [14].

The multimodal treatment strategy for castration-sensitive metastatic prostate cancer involves the initiation of ADT according to current guidelines [15]. In the context of SNRT, ADT may be delayed to avoid ADT-related side effects [4]. The data from randomized trials, which evaluate the combination of ADT and SNRT, are not available yet. Therefore, in this current analysis, we investigated the long-term outcome and toxicity of salvage radiotherapy with or without ADT for nodal oligorecurrent prostate cancer with PET-guided SIB to involved lymph nodes (LN).

## 2. Materials and Methods

This retrospective single-center analysis is based on 95 consecutive patients treated between 2010 and 2021 with SNRT for (oligo)recurrent prostate cancer after initial resection and/or radiotherapy. All patients had histologically proven prostate cancer. Stratification was conducted according to the risk group classification of D’Amico et al. [16]. PET imaging before SNRT was conducted with ^68^Ga-labeled PSMA PET in 45 cases (47.4%), and with ^18^F-PSMA-1007 PET in 22 cases (23.2%). A total of 23 patients (24.2%) received ^18^F-Fluoromethylcholine PET, 4 patients (4.2%) received ^11^C-Choline PET, and one patient (1.1%) received conventional imaging as the pre-treatment staging method. 

**Figure 1:**
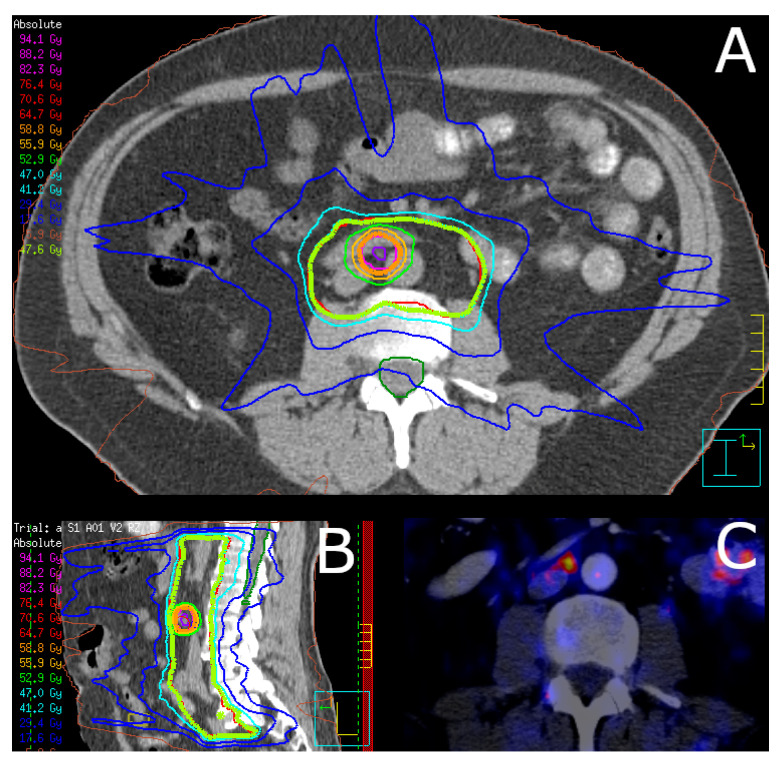
Exemplary dose distribution of salvage nodal radiotherapy. Shown is an exemplary dose distribution of a patient receiving PSMA PET-guided salvage nodal radiotherapy in (**A**) axial view and (**B**) sagittal view. The corresponding ^68^Ga-labeled PSMA PET shows a single paraaortic lymph node metastasis (**C**). The patient received volumetric modulated arc therapy with a simultaneous integrated boost to the involved PET-positive lymph node. Twenty-eight fractions with 1.7 Gy per fraction for the PTV and 2.1 Gy per fraction for the PTV_Boost_ were prescribed, resulting in total doses of 47.6 Gy and 58.8 Gy, respectively.

SNRT was delivered by intensity-modulated radiation therapy (IMRT) or volumetric modulated arc therapy (VMAT) with SIB to recurrent PET-positive LN. Concomitant ADT was prescribed at the discretion of the treating urologist and was recommended for patients with an intermediate-risk (for 6 months) and a high-risk disease (for 24–36 months). A total of 79 (83.2%) patients received SNRT without irradiation of the prostatic fossa. Cone-beam computed tomography-guided SNRT was moderately hypofractionated with five fractions per week. Two dose levels were applied in the SIB technique: The prescribed median PTV dose (D_95%_) was 47.6 Gy (interquartile range (IQR) 45.9–49.3 Gy) in 28 fractions (IQR 27–28 fractions) of 1.7 Gy per fraction (IQR 1.7–1.8 Gy). The prescribed median total PTV_Boost_ dose (D_mean_) to recurrent lymph nodes was 60.2 Gy (IQR 58.8–63.0 Gy) with a median dose per fraction of 2.2 Gy (IQR 2.1–2.2 Gy). Prophylactic irradiation of the prostatic fossa without signs of recurrence in PET imaging was conducted in 16 (16.8%) cases. The prescribed median total dose to the prostatic fossa was 69.3 Gy (IQR 57.6–69.3 Gy) with a median dose per fraction of 2.1 Gy (IQR 2.1–2.1 Gy) in 33 fractions (IQR 29–33 fractions). Target volume delineation was based on the Radiation Therapy Oncology Group (RTOG) atlas for salvage prostate cancer and was adjusted at the discretion of the treating radiation oncologist, especially for extrapelvic disease. In 65 (68.4%) cases the pelvic, in 18 (18.9%) cases the extrapelvic, and in 12 (12.6%) cases the pelvic plus, extrapelvic LN were treated. Pinnacle^3^ (Philips Radiation Oncology Systems, Fitchburg, WI, USA) was used for treatment planning. Figure 1 illustrates an exemplary dose distribution.

Biochemical progression was the primary endpoint of this retrospective study and was defined as nadir plus a ≥ 0.5 ng/mL increase in the PSA value in accordance with the GETUG AFU 16 trial definition [17]. Secondary endpoints were metastatic disease progression, overall mortality, and the five-year cumulative incidence of gastrointestinal and genitourinary toxicity. Metastastic disease progression was defined as the occurrence of distant metastasis, diagnosed by follow-up imaging. The occurrence of death from any cause was defined as overall mortality. The follow-up was defined as the time period between the start of radiotherapy and the date of the last follow-up. Physician-recorded toxicity was assessed at baseline, at the end of radiotherapy, six weeks after radiotherapy, and then every six months. Follow-up was changed to annual examinations after the first two years of follow-up. Gastrointestinal (GI) and genitourinary (GU) side effects were assessed utilizing Common Terminology Criteria for Adverse Events (CTCAE) v5.0 [18]. Side effects, which occurred between the start of SNRT and three months after SNRT, were counted as acute toxicity, whereas all later follow-ups were included in the late toxicity assessment.

Cumulative incidence functions (CIFs) with the start of radiation therapy set as the baseline time were used to generate five-year outcome estimates. CIFs were used for biochemical and metastatic disease progression with death as the competing risk (R-package cmprsk). For overall mortality, no competing risk was assumed. Gray´s tests were used to compare cumulative incidence estimates. The impact of clinico-pathological parameters on biochemical and metastatic disease progression was assessed by uni- and multivariate Fine–Gray competing-risk regression analyses, considering as covariates extrapelvic lymph nodes (no/yes), number of lymph nodes ≥ 4 (no/yes), PSMA PET (no/yes), age at start of radiotherapy (continuous), Gleason score ≥ 8 (no/yes), concomitant ADT (no/yes), and PSA value at the start of radiotherapy (continuous). The Fine–Gray models were fit to estimate the incidence of outcomes over time in the presence of competing risks (finegray, R-package survival). The Fine–Gray model fits were generated by fitting a weighted Cox model on the constructed Fine–Gray data set, as described by Therneau et al. [19]. The best fitting weighted Cox model was selected by measuring the relative goodness-of-fit with the Akaike information criterion (AIC) (stepAIC, R-package MASS), which selected a combination of patient ages at the start of radiotherapy, concomitant ADT, and PSA value at the start of radiotherapy as the best predictors for biochemical progression. The covariates number of lymph nodes ≥ 4, PSMA PET, concomitant ADT, and PSA value at the start of radiotherapy were selected as the best predictors for metastatic disease progression. The model fits were assessed by comparing the results of the likelihood ratio tests. The proportional hazard assumption was tested by analyzing the Schoenfeld residuals (cox.zph, R-package survival). For all statistical analyses, R version 4.2.0 (The R Foundation, Vienna, Austria) was utilized. All tests were two-sided with statistical significance indicated by *p* < 0.05.

## 3. Results

The median follow-up was 47.1 months in total (IQR 24.3–75.0 months), and it was not significantly different between ADT and non-ADT groups. The prescribed median equivalent dose (EQD2_1.5Gy_) to the recurrent lymph node metastases was 62.9 Gy (IQR 60.5–66.9 Gy), and the median EQD2_1.5Gy_ to the PTV was 43.5 Gy (IQR 42.0–46.1 Gy). Prophylactic prostatic fossa irradiation amounted to a prescribed median equivalent dose (EQD2_1.5Gy_) of 71.3 Gy (IQR 59.4–71.3 Gy). The clinical characteristics of the cohort are summarized in Table 1.

The estimated five-year biochemical progression rate was 50.1% (95% CI: 35.7–62.9%). The estimated five-year metastatic disease progression rate reached 36.8% (95% CI: 24.0–49.7%), and the five-year overall mortality rate was 9.1% (95% CI: 3.1–19.0). In total, 34 cases (35.8%) relapsed biochemically. A total of 26 cases (27.4%) had distant metastasis and 15 cases (15.8%) had died of any cause during follow-up. In all 26 cases of distant metastasis, defined as non-regional lymph node relapse or metastasis, relapse was out-of-field. Local relapse in the prostatic fossa was observed in six cases (6.3%), out of which one case relapsed in-field and five cases out-of-field. Regional relapse in the pelvic lymph nodes occurred in 13 cases (13.7%), out of which 4 cases relapsed in-field and 9 cases out-of-field.

**Figure 2:**
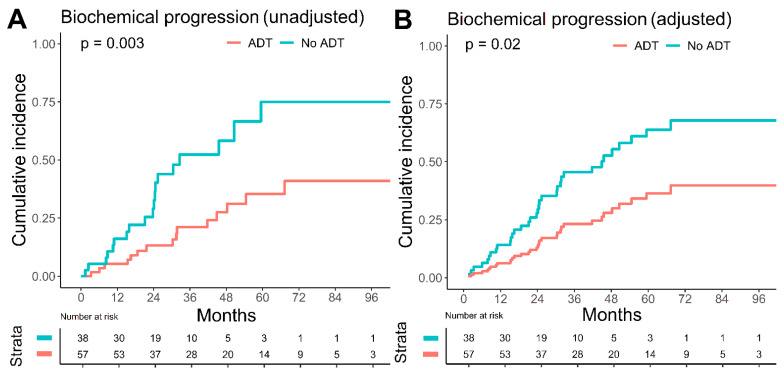
Influence of androgen deprivation therapy on biochemical progression. Cumulative incidence of biochemical progression, stratified by concomitant androgen deprivation therapy (ADT), with death as competing risk. Plot (**A**) shows the unadjusted biochemical progression, whereas plot (**B**) demonstrates the biochemical progression after adjustment for confounders by Fine–Gray regression analysis. Biochemical progression was significantly lower in the group receiving concomitant ADT (red line) versus without ADT (turquoise line).

Concomitant ADT was adminstered in 60% of the patients with a median duration of 25.4 months (IQR 19.7–45.3 months). Biochemical progression was significantly higher in the group receiving no ADT with an estimated five-year biochemical progression rate of 75.0% (95% CI: 42.0–90.9%) versus 35.3% (95% CI: 19.6–51.4%) with ADT (*p* = 0.003), Figure 2. In the group with up to three LN (n = 75), the biochemical progression rate was also significantly lower if concomitant ADT was given (five-year biochemical progression rate with ADT 69.6% versus 26.2% without ADT, *p* = 0.001). No significant influence of ADT on metastatic disease progression and overall mortality was observable.

After adjustment for confounders, concomitant ADT was still significantly associated with reduced biochemical progression in the multivariate Fine–Gray regression analysis with all covariates (HR 0.41, 95% CI: 0.19–0.86, *p* = 0.02), as well as in the multivariate analysis utilizing the AIC (HR 0.42, 95% CI: 0.21–0.84, *p* = 0.01), which selects the best fitting model out of a set of considered covariates. Concomitant ADT was also prognostic for reduced metastatic disease progression in the multivariate model with all covariates (HR 0.36, 95% CI: 0.15–0.85, *p* = 0.02), as well as in the multivariate model with the best AIC (HR 0.39, 95% CI: 0.17–0.90, *p* = 0.03). Compared to unadjusted HR, after it has been controlled for patient age and PSA value at the start of radiotherapy, the protective effect of ADT in biochemical progression weakened from 0.39 (95% CI: 0.19–0.76) to 0.42 (95% CI: 0.21–0.84), but the statistical significance improved from *p* = 0.07 to *p* = 0.01. In metastatic disease progression, the protective effect became stronger after being adjusted by the PSA value at the RT start, number of lymph nodes, and the PET imaging method, with HR having improved from 0.54 (95% CI: 0.25–1.17) to 0.39 (95% CI: 0.17–0.90), as well as the *p*-value from 0.1 to 0.03. The PSA value at the start of radiotherapy reached significance in the multivariate model with the best AIC (HR 1.04, 95% CI: 1.01–1.08) for increased metastatic disease progression. The results of the uni- and multivariate Fine–Gray regression analyses are summarized in Table 2.

**Figure 3:**
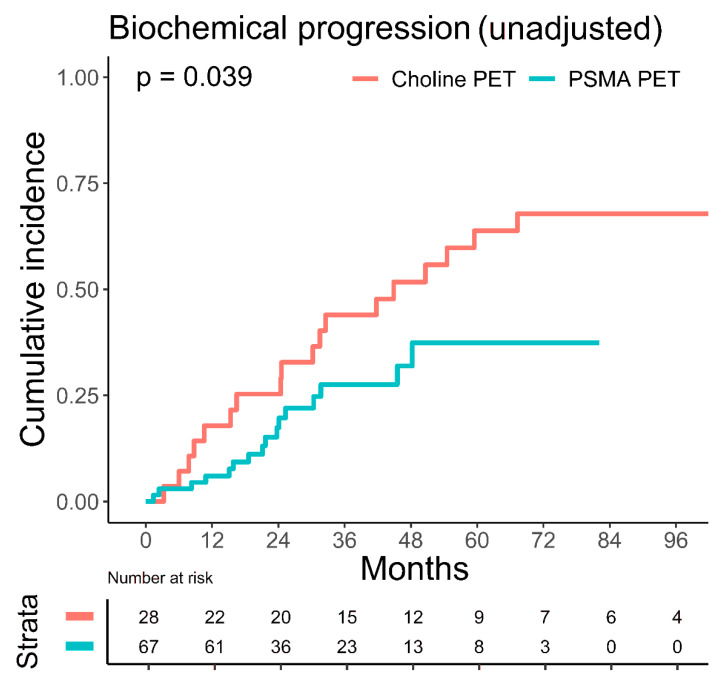
Influence of the PET imaging method on biochemical progression. Cumulative incidence of biochemical progression, stratified by the PET imaging method (PSMA versus choline), with death as competing risk. Unadjusted, estimated five-year biochemical progression rate was significantly lower with 37.4% (95% CI: 20.6–54.2%) for PSMA PET (turquoise line) versus 63.8% (95% CI: 41.6–79.4%) for choline PET (red line), *p* = 0.039 (Gray´s test).

The unadjusted, estimated five-year biochemical progression rate was significantly lower in patients imaged with PSMA PET with 37.4% (95% CI: 20.6–54.2%) versus 63.8% (95% CI: 41.6–79.4%) in patients imaged with choline PET (*p* = 0.039), Figure 3. Metastatic disease progression and overall mortality were not significantly different between patients imaged with choline or PSMA PET. PET imaging method did not significantly influence biochemical progression in multivariate Fine–Gray regression analysis (Table 2).

The median number of PET-positive recurrent lymph nodes (LN) was 1 LN (IQR 1–3 LN). The number of PET-positive LN metastases had a significant influence on overall mortality, but not on the biochemical progression and metastatic disease progression: the estimated five-year overall mortality rate was 3.1% (95% CI: 0.2–14.0%) for 1–3 LN versus 28.1% (95% CI: 7.5–53.7%) for ≥4 LN (*p* = 0.027).

Acute GU grade 2 toxicity occurred in 16.8% (n = 16) and acute GI grade 2 toxicity in 5.3% (n = 5). No cases of grade 3 or higher acute toxicity were observable. A cumulative three-year and five-year late GU ≥ grade 2 toxicities were seen in 14.7% (n = 14) and 15.8% (n = 15) of cases, respectively. A cumulative three-year and five-year late GI ≥ grade 2 toxicities were both 1.1% (n = 1). A cumulative three-year and five-year late grade 3 GU toxicity each occurred in 2.1% (n = 2) of cases. Late grade 3 GU toxicity consisted of two patients with urinary incontinence, out of which one patient received prophylactic prostate fossa radiotherapy. Late grade 3 GI toxicity did not occur.

## 4. Discussion

Metastasis-directed therapy in oligometastatic prostate cancer has recently gained attention based on the available data from the STOMP and ORIOLE trials, which showed a benefit for MDT in the oncological outcome [4,5]. The present analysis demonstrates an estimated five-year biochemical progression rate of 50.1% (95% CI: 35.7–62.9%), a five-year metastatic disease progression rate of 36.8% (95% CI: 24.0–49.7%), and an overall mortality rate of 9.1% (95% CI: 3.1–19.0) for SNRT in nodal oligorecurrent prostate cancer. As the overall cohort suffered from unfavorable disease characteristics with ≥4 LN metastases in 20% and with LN metastases located outside of the pelvis (stage M1a) in >30% of all patients, the oncological outcome is encouraging. Our data compare favorably to the literature: Fodor et al. reported a three-year biochemical relapse-free survival (BRFS) of 42.4% in a group of 81 patients with SNRT [20]. Ingrosso et al. published a three-year BRFS of 53% in 41 patients [21]. Both studies utilized choline PET as the staging method whereas the current standard of care, PSMA PET imaging, was preferentially utilized in the present work. In a more recent analysis by Rogowski et al., 100 patients received SNRT after resection and nodal recurrence, detected by PSMA PET [22]. The reported three-year BRFS of 65.8% is comparable to our data (three-year biochemical progression rate of 33.4%). The toxicity data were not reported by Rogowski et al., but SNRT was safe in our cohort with a cumulative three-year late grade 3 GU toxicity rate of 2.1% (n = 2) and no cases of late grade 3 GI toxicity.

In patients with nodal oligorecurrent prostate cancer, SNRT may improve clinically important endpoints and present a chance to delay disease progression [14]. In our analysis, patients with up to three LN metastases showed lower estimated five-year overall mortality compared to patients suffering from more than three LN metastases, with 3.1% (95% CI: 0.2–14.0%) versus 28.1% (95% CI: 7.5–53.7%), *p* = 0.027. PET imaging facilitates the decision-making process by distinguishing limited nodal oligorecurrent prostate cancer from multi-metastatic disease [23]. In a recent meta-analysis, the performance of PSMA and choline PET was compared in prostate cancer biochemical recurrence: PSMA PET showed a superior pooled detection rate over choline PET with 44% versus 24% for PSA levels less than 0.5 ng/mL [24]. Bluemel et al. investigated sequential ^68^Ga-PSMA PET after negative ^18^F-Choline PET in 139 patients [25]. ^68^Ga-PSMA PET was able to identify sites of recurrent disease in 43.8% of the patients with negative ^18^F-Choline PET scans. In our analysis, the unadjusted, estimated five-year biochemical progression rate was significantly higher in patients imaged with choline PET with 63.8% (95% CI: 41.6–79.4%) versus 37.4% (95% CI: 20.6–54.2%) in patients imaged with PSMA PET (*p* = 0.039). In multivariate Fine–Gray regression analysis, the PET imaging method did not significantly influence outcome. Nonetheless, the higher detection rate of PSMA PET for low PSA levels may result in the earlier detection of oligometastatic disease and the earlier initiation of SNRT and, therefore, an improved outcome. Modern, state-of-the-art imaging methods are needed to distinguish curable cases. In the case of multi-metastatic burden, systemic treatment may be preferred over SNRT [15]. Nonetheless, systemic treatment may affect quality of life due to treatment-related toxicity. For oligorecurrent prostate cancer, SNRT, therefore, aims at deferring ADT usage to reduce ADT-related toxicity and improve quality of life without compromising the oncologic outcome [15]. Ost et al. investigated the benefit in ADT-free survival by MDT in oligorecurrent prostate cancer and showed an improvement in ADT-free survival for the MDT group (13 versus 21 months) [4]. Currently, no consensus exists in which cases of ADT could be safely delayed in favor of MDT without compromising the oncologic outcome. ADT represents the standard of care for metastatic prostate cancer, according to the current guidelines of the European Association of Urology [15]. In our study, with an estimated five-year biochemical progression rate of 75.0% (95% CI: 42.0–90.9%) without ADT versus 35.3% (95% CI: 19.6–51.4%) with ADT (*p* = 0.003), additional ADT significantly reduced biochemical progression for patients receiving SNRT. The improvement in biochemical progression persisted if only patients with up to three oligometastases were analyzed. Furthermore, after adjustment for confounders, ADT was still significantly influencing biochemical progression (HR 0.42, 95% CI: 0.21–0.84, *p* = 0.01) and metastatic disease progression (HR 0.39, 95% CI: 0.17–0.90, *p* = 0.03). ADT was independent from PSMA PET imaging in Fine–Gray regression analysis (Table 2). In the OLIGOPELVIS GETUG P07 phase 2 trial, 67 patients received short-term ADT (for 6 months) and SNRT, resulting in an encouraging three-year BRFS rate of 46% [26]. Moreover, in a retrospective analysis of 305 patients, Kroeze et al. observed improved BRFS with ADT in addition to SNRT in oligorecurrent prostate cancer patients with a two-year BRFS of 78% versus 53% (*p* < 0.0001) [27]. A limitation of the analysis of Kroeze et al. is the short median follow-up of 16 months, whereas the follow-up in the current work reached a median of 47.1 months.

The results of ongoing prospective studies will further add to the evidence for the combination of ADT and MDT as a multimodal treatment strategy. The phase 3 OLIGOPELVIS 2 GETUG P12 trial investigates short-term ADT with or without SNRT (NCT03630666), whereas the phase 2 PEACE V (STORM) trial compares salvage MDT with SABR or salvage resection + ADT versus MDT + whole pelvis RT + ADT (NCT03569241) [28]. The randomized phase 3 ADOPT trial aims at assessing the benefit of the addition of 6 months of ADT to MDT in a PSMA PET-staged cohort of patients with one to four recurrent oligometastatic lesions [29].

Salvage lymph node resection (SLND) as a competing form of MDT for oligorecurrent prostate cancer has been explored. Farolfi et al. used repeated PSMA PET to detect PET-positive lesions after unsuccessful SLND. Notably, after SLND, two-thirds of the patients had PET-positive nodal disease, which was already observable on pre-SLND PSMA PET. This challenges the concept of SLND as a monotherapy for oligorecurrent prostate cancer [30,31]. In a recent retrospective analysis comparing PSMA PET-staged unilateral SLND versus SNRT, the biochemical recurrence rate at maximum follow-up was 40.3% (27 patients) for the SNRT cohort and 86.4% (57 patients; *p* < 0.001) for the SLND cohort [32]. Due to the heterogeneity of both groups and the lack of bilateral SLND, no definitive conclusions can be drawn regarding the superiority of one form of MDT over the other. Data from prospective, randomized trials are not yet available and are eagerly awaited.

A limitation of the present study is the relatively small number of patients and the inherent heterogeneity in the patient characteristics as well as the treatment parameters attributable to the retrospective nature of the conducted analysis. Randomized controlled trials are needed to further elucidate the role of SNRT in nodal oligorecurrent prostate cancer.

## 5. Conclusions

In this work, we investigated metastasis-directed therapy in the form of salvage nodal radiotherapy for PET-staged oligorecurrent prostate cancer in a group of 95 consecutive patients. Cumulative incidence functions and Fine–Gray regression analyses were applied to investigate the outcome. Overall, salvage nodal radiotherapy demonstrated encouragingly low rates of long-term biochemical progression, metastatic disease progression, overall mortality, and toxicity, which supports the use of metastasis-directed therapy for oligometastatic prostate cancer.

The addition of concomitant androgen deprivation therapy to salvage nodal radiotherapy led to significantly decreased biochemical progression. Androgen deprivation therapy was also prognostic for lower biochemical and metastatic disease progression in multivariate confounder-adjusted analyses. The combination of salvage nodal radiotherapy with simultaneous integrated boost and androgen deprivation therapy in patients with nodal oligorecurrent prostate cancer warrants further investigation in randomized controlled trials.

## Figures and Tables

**Table 1:** Clinical characteristics.

Characteristics	Overall	No ADT	ADT	*p* Value
**Patients, n (%)**	95 (100)	38 (40)	57 (60)	
**Follow-up (months)**	47.1 (24.3, 75.0)	45.7 (24.6, 70.3)	50.4 (23.6, 79.9)	0.605
**KPS (%)**	90 (90, 100)	100 (90, 100)	90 (90, 100)	0.248
**Age at SNRT start (years)**	70.6 (66.3, 75.0)	70.4 (63.8, 74.5)	72.3 (67.9, 75.2)	0.364
**PSA at primary diagnosis (ng/mL)**	10.9 (6.7, 17.7)	8.9 (6.5, 14.0)	11.4 (7.4, 24.6)	0.102
**PSA at primary diagnosis, n (%)**				0.120
<10 ng/mL	45 (47.4)	21 (55.3)	24 (42.1)	
10–20 ng/mL	26 (27.4)	11 (28.9)	15 (26.3)	
>20 ng/mL	20 (21.1)	4 (10.5)	16 (28.1)	
N/A	4 (4.2)	2 (5.3)	2 (3.5)	
**PSA at SNRT start (ng/mL)**	2.3 (0.6, 4.9)	1.44 (0.5, 3.8)	3.0 (0.7, 5.3)	0.272
**Gleason-Score**				0.977
≤6	13 (13.7)	5 (13.2)	8 (14.0)	
7	45 (47.4)	18 (47.4)	27 (47.4)	
≥8	36 (37.9)	15 (39.5)	21 (36.8)	
N/A	1 (1.1)	0 (0)	1 (1.8)	
**Initial stage, n (%)**				0.576
≤T2a	11 (11.6)	6 (15.8)	5 (8.8)	
T2b	5 (5.3)	2 (5.3)	3 (5.3)	
≥T2c	79 (83.2)	30 (78.9)	49 (86.0)	
**Initial D’Amico risk class, n (%)**				0.229
Low	3 (3.2)	2 (5.3)	1 (1.8)	
Intermediate	6 (6.3)	4 (10.5)	2 (3.5)	
High	86 (90.5)	32 (84.2)	54 (94.7)	
**Initial treatment before SNRT**				0.629
Surgery only	33 (34.7)	11 (28.9)	22 (38.6)	
Surgery + adjuvant RT	15 (15.8)	8 (21.1)	7 (12.3)	
Surgery + salvage RT	35 (36.8)	14 (36.8)	21 (36.8)	
Primary RT	12 (12.6)	5 (13.2)	7 (12.3)	
**PET imaging, n (%)**				0.480
PSMA PET	67 (70.5)	29 (76.3)	38 (66.7)	
Choline PET	27 (28.4)	9 (23.7)	18 (31.6)	
Conventional	1 (1.1)	0 (0)	1 (1.8)	
**PET-positive lymph node metastases, n (%)**				0.476
1	49 (51.6)	21 (55.3)	28 (49.1)	
2	21 (22.1)	12 (31.6)	9 (15.8)	
3	5 (5.3)	2 (5.3)	3 (5.3)	
≥4	20 (21.1)	3 (7.9)	17 (29.8)	
**SNRT location**				0.253
Pelvic (N1)	65 (68.4)	30 (78.9)	35 (61.4)	
Extrapelvic (M1a)	18 (18.9)	6 (15.8)	12 (21.1)	
Pelvic + extrapelvic (N1 + M1a)	12 (12.6)	2 (5.3)	10 (17.5)	

Abbreviations: ADT = androgen deprivation therapy; KPS = Karnofsky performance score; N/A = not available; PET = positron emission tomography; PSA = prostate-specific antigen; PSMA = prostate-specific membrane antigen; RT = radiotherapy; SNRT = salvage nodal radiotherapy. Estimates are given as median (quartile 1, quartile 3) or frequency (percentage). *p* values were calculated using Mann-Whitney-U test for continuous and χ^2^ test for categorical variables.

**Table 2:** Uni- and multivariate Fine–Gray competing-risk regression analyses.

Biochemical Progression	Univariate		Multivariate		Multivariate AIC	
Variables	HR (95% CI)	*p* Value *	HR (95% CI)	*p* Value *	HR (95% CI)	*p* Value *
**PSA at RT start**	1.05 (1.02–1.08)	0.03	1.02 (0.96–1.09)	0.44	1.04 (1.00–1.08)	0.05
**Number of LN ≥ 4**						
No	Ref		Ref			
Yes	1.39 (0.66–2.96)	0.40	1.49 (0.56–3.96)	0.42		
**Gleason score ≥ 8**						
No	Ref		Ref			
Yes	1.00 (0.49–2.04)	1.00	1.17 (0.53–2.58)	0.70		
**Age at start of RT**	1.05 (1.00–1.10)	0.06	1.04 (0.99–1.10)	0.09	1.05 (1.00–1.10)	0.06
**Concomitant ADT**						
No	Ref		Ref		Ref	
Yes	0.39 (0.19–0.76)	0.07	0.41 (0.19–0.86)	0.02	0.42 (0.21–0.84)	0.01
**Extrapelvic disease**						
No	Ref		Ref			
Yes	1.08 (0.54–2.14)	0.80	0.89 (0.33–2.39)	0.82		
**PET imaging**						
Choline PET	Ref		Ref			
PSMA PET	0.52 (0.26–1.03)	0.06	0.62 (0.29–1.33)	0.22		
**Metastatic disease progression**	**Univariate**		**Multivariate**		**Multivariate AIC**	
**Variables**	**HR (95% CI)**	*p* Value *	**HR (95% CI)**	*p* Value *	**HR (95% CI)**	*p* Value *
**PSA at RT start**	1.05 (1.01–1.09)	0.02	1.03 (1.00–1.07)	0.07	1.04 (1.01–1.08)	0.02
**Number of LN ≥ 4**						
No	Ref		Ref		Ref	
Yes	2.12 (0.94–4.77)	0.10	2.02 (0.68–5.98)	0.20	2.35 (0.92–6.03)	0.07
**Gleason score ≥ 8**						
No	Ref		Ref			
Yes	0.73 (0.32–1.69)	0.50	0.90 (0.36–2.24)	0.82		
**Age at start of RT**	1.02 (0.98–1.06)	0.50	1.00 (0.95–1.04)	0.91		
**Concomitant ADT**						
No	Ref		Ref		Ref	
Yes	0.54 (0.25–1.17)	0.10	0.36 (0.15–0.85)	0.02	0.39 (0.17–0.90)	0.03
**Extrapelvic disease**						
No	Ref		Ref			
Yes	1.83 (0.86–3.90)	0.10	1.61 (0.53–4.91)	0.40		
**PET imaging**						
Choline PET	Ref		Ref		Ref	
PSMA PET	0.62 (0.26–1.45)	0.20	0.53 (0.24–1.16)	0.11	0.55 (0.24–1.24)	0.15

* Likelihood ratio test. For biochemical progression, multivariate global *p*-value reached 0.030 and improved to 0.004 by employing the Akaike information criterion (AIC). For metastatic disease progression, multivariate global *p*-value improved from 0.060 to 0.010 by utilizing AIC. Bold *p* values indicate statistically significant results. Abbreviations: AIC = Akaike information criterion; ADT = androgen deprivation therapy; CI = confidence interval; HR = hazard ratio; LN = lymph node; PET = positron emission tomography; PSA = prostate-specific antigen; PSMA = prostate-specific membrane antigen; Ref = reference; RT = radiotherapy.

## Data Availability

The data presented in this study are available on request from the corresponding author. The data are not publicly available due to privacy restrictions.
